# Beyond Uptake in Cancer Lipid Nanomedicines: Linking Tumour Access, Intracellular Fate, and Molecular Action

**DOI:** 10.3390/pharmaceutics18101212

**Published:** 2026-09-24

**Authors:** Syed Alhafiz Syed Hashim

**Affiliations:** Department of Pharmacology, Faculty of Medicine, Universiti Kebangsaan Malaysia, Kuala Lumpur 56000, Malaysia; syedalhafiz@ukm.edu.my

**Keywords:** lipid nanoparticles, liposomes, cancer nanomedicine, endosomal escape, intracellular trafficking, functional delivery, protein corona, transcytosis, RNA therapeutics, target engagement

## Abstract

Clinical translation of lipid nanomedicines in oncology is limited by biological barriers and by an interpretive challenge: tumour accumulation and cellular uptake are often used as proxies for delivery even when pharmacologically competent cargo, meaning cargo capable of producing its intended molecular effect, has not reached the relevant cell and intracellular machinery required for its intended molecular action. This narrative review synthesises mechanistic and translational evidence across liposomes, ionisable lipid nanoparticles (LNPs), cationic lipid systems, solid lipid platforms, and lipid–polymer hybrids, focusing on systemically administered formulations for solid tumours. The review follows the delivery sequence from tumour access and cell-type allocation (distribution of tumour-accessed material among tumour-associated cell populations) through internalisation, intracellular sorting, endosomal escape where required, cargo release or bioconversion, and cargo-specific molecular action. Upstream gains can fail downstream through sequestration, recycling, lysosomal trapping, inefficient escape, delayed release, uncertain cell allocation, or toxicity. Because delivery requirements are cargo-dependent, nucleic-acid LNPs generally require cytosolic access and intracellular machinery, whereas liposomal small molecules may depend more on timed release, bioconversion, and active-drug exposure. Strong mechanistic inference therefore requires readouts that distinguish carrier from cargo, resolve cellular and subcellular location, and demonstrate cargo-specific pharmacology. The central design implication is to identify the dominant biological bottleneck and prioritise safe, cell-specific pharmacological output rather than uptake intensity alone.

## 1. Introduction: Why Uptake Is Not Equivalent to Delivery

The central challenge in cancer drug delivery is not simply to identify a potent cargo, but to convert an administered dose into sufficient molecular action in the relevant malignant, stromal, or immune cell while preserving a viable therapeutic index. Lipid-based nanomedicines can protect labile nucleic acids, alter systemic pharmacokinetics, reduce exposure to vulnerable normal tissues, and enable delivery of membrane-impermeable macromolecules. Their performance remains strongly conditioned by biological access, cell allocation, and intracellular processing; solid tumours add vascular heterogeneity, stromal competition, and intracellular attrition before pharmacology can occur [1,2,3,4].

A persistent weakness in delivery studies is mechanistic linearity: tumour accumulation is treated as evidence of tumour penetration; penetration as evidence of target-cell access; cell association as internalisation; and internalisation as cytosolic delivery. Each inference can fail. Whole-tumour fluorescence may largely reflect intravascular, perivascular, stromal, or phagocytic sequestration. Flow-cytometric signal can include surface-bound particles. Intracellular fluorescence may represent material trapped in endosomes or lysosomes. Galectin recruitment reports membrane damage rather than intact cargo translocation, and even cytosolic cargo is not equivalent to target engagement. These distinctions separate a pharmacokinetic observation from a therapeutic mechanism [5,6,7,8,9,10].

Two influential studies made this disconnect difficult to ignore. Gilleron and colleagues used quantitative imaging and electron microscopy to show that only a small fraction of LNP-delivered small interfering ribonucleic acid (siRNA) escaped endosomes in the system examined [5]. Sahay and colleagues showed that Niemann–Pick C1 (NPC1)-dependent recycling could remove a large fraction of internalised siRNA-LNP material and thereby limit functional availability [6]. Live-cell studies subsequently linked siRNA release to a brief window during endosomal maturation and showed that relatively few cytosolic siRNA molecules can be sufficient for gene silencing [7]. Together, these studies establish intracellular fate—not uptake alone—as a major determinant of conversion from dose to pharmacology.

The translational problem begins even earlier. Across heterogeneous nanoparticle datasets, only a small fraction of intravenously administered material reaches solid tumours, and the fraction that reaches the intended malignant cell population is still smaller [4,11]. At the tumour vessel, evidence supports both paracellular leakage and active endothelial transport, with their relative contributions varying by particle, tumour, vascular phenotype and experimental model [2,3,12,13,14]. Therefore, no single upstream metric—circulation half-life, percentage injected dose per gram, total cell-associated fluorescence or internalisation rate—can serve as a universal proxy for successful delivery.

This narrative review examines how an administered lipid nanomedicine is converted into a pharmacologically active therapeutic species in the relevant tumour-associated cell. The discussion follows established biological processes from tissue access and cell allocation to intracellular trafficking, cargo release or bioconversion, and molecular action. The aim is to distinguish what each experimental readout actually demonstrates, prevent upstream observations from being used as proof of downstream mechanisms, and connect mechanistic evidence to clinically relevant pharmacology.

The review is organised around the biological transitions at which delivery can be gained or lost. Tumour accumulation, target-cell allocation, internalisation, intracellular routing, endosomal escape, cargo release, and target engagement are treated as experimentally distinct processes because improvement in one does not guarantee improvement in the next. Mechanistic claims are considered strongest when localization is supported by causal perturbation or orthogonal validation, cargo-specific molecular action, and evidence from a biological context appropriate to the translational question.

The following two questions therefore organise this narrative review: which biological step currently limits pharmacological output, and what evidence shows that an intervention improved that step without sacrificing downstream function or safety?

## 2. Scope and Approach of This Narrative Review

This mechanism-centred narrative review focuses on systemically administered lipid nanomedicines for solid tumours, with greatest emphasis on ionisable LNPs and liposomes because these platforms provide the richest mechanistic and clinical evidence. Permanently cationic systems, solid lipid nanoparticles (SLNs)/nanostructured lipid carriers (NLCs), and lipid–polymer hybrids are included where they expose platform-specific differences in uptake, trafficking, release, or toxicity. Hepatic and non-oncology LNP studies are used selectively when they establish general principles of biological identity, intracellular routing, endosomal escape, or functional delivery that can inform—without being assumed to reproduce—tumour delivery.

No single solid-tumour histology was prespecified; evidence from diverse solid-tumour models and clinical settings was considered where it informed the delivery transitions examined. The review focuses on systemic administration; local or intratumoural delivery was outside the primary scope. The relevant literature was identified through targeted searches of PubMed, Scopus, and Google Scholar, supplemented by reference-list screening and citation tracking of key articles.

The literature is organised by mechanistic transition rather than chronologically. Priority is given to studies that resolve the denominator of a delivery claim, distinguish carrier from cargo, provide causal or compartment-resolved evidence, or connect delivery to cargo-specific pharmacology and human translation. Because the purpose of this review is conceptual and mechanism-centred rather than systematic evidence synthesis, studies were selected purposively for their ability to illustrate, challenge, or delimit specific transitions in lipid nanomedicine delivery. Particular emphasis was placed on mechanistically informative primary studies, clinically relevant examples, and reports that expose discrepancies between upstream delivery surrogates and downstream pharmacological function. Seminal studies were included where they established concepts that remain central to current interpretation, while recent studies were used where they refine, challenge, or extend those concepts.

## 3. From Systemic Exposure to Molecular Action: A Mechanistic Sequence

For systemically administered lipid nanomedicines, therapeutic activity depends on a sequence of established biological events as follows: circulation and tumour access, distribution among tumour-associated cell populations, internalisation where required, intracellular sorting, endosomal escape for cargos that require cytosolic access, cargo release or bioconversion, and cargo-specific target engagement. The relative importance of these events differs by platform and payload. Earlier multistage descriptions, such as the circulation, accumulation, penetration, internalisation, and release (CAPIR) cascade, remain useful for organising extracellular-to-cell delivery barriers [15], while the cancer gene-delivery literature has long emphasised additional intracellular constraints [16]. In this narrative review, these established processes are used to organise the mechanistic evidence across lipid nanomedicine classes.

Each experimental readout answers a different biological question. Increased whole-tumour accumulation does not establish improved delivery to malignant cells; increased cell-associated fluorescence does not establish internalisation; internalisation does not establish favourable intracellular sorting; and endosomal membrane damage does not prove cytosolic translocation of intact, functional cargo. The interpretation therefore depends on the denominator and biological compartment represented by the measurement, as well as on whether the carrier and cargo remain associated at the time of analysis.

The relevant route is also cargo-specific. Nucleic-acid LNPs commonly require cellular entry, escape from endosomal compartments, release of intact ribonucleic acid (RNA), and access to RNA-induced silencing complex (RISC), ribosomes, or genome-editing machinery. By contrast, liposomal small-molecule drugs can derive benefit from altered pharmacokinetics and local drug release without cytosolic entry of the intact carrier. Prodrugs and bioconversion-dependent therapies add another requirement: formation and persistence of the active species. These differences make it inappropriate to judge all lipid nanomedicines by a single uptake or escape metric.

A common experimental paradox follows from this biology: a larger uptake signal can coexist with lower pharmacological output when a formulation redirects material toward recycling, lysosomal sequestration, damaging membrane disruption, or delayed cargo release. Conversely, modest internalisation can outperform extensive uptake when a larger fraction of cargo reaches the required compartment and remains functionally competent. This decoupling of uptake, escape, and functional expression is observed across multiple delivery systems and assays [5,6,7,8,9,10,17,18,19,20].

Formulation screening should pair an exposure or cell-allocation measurement with a compartment-resolved or cytosolic readout and a cargo-specific functional endpoint. Ranking particles by bulk fluorescence alone privileges an upstream signal while leaving downstream attrition unmeasured. The published examples below illustrate how mechanistic interpretation changes when downstream function is considered alongside uptake. These distinctions are summarised in the graphical abstract, which separates tumour-level biodistribution/access, cellular uptake and allocation, intracellular delivery, and cargo-specific pharmacological activity.

### Illustrative Studies: When Uptake and Function Diverge

The examples in Table 1 span intracellular attrition, tumour access, clinical RNA delivery, and small-molecule liposomes. They are not intended as an exhaustive evidence synthesis; rather, they illustrate recurring mechanistic mismatches between upstream delivery signals and downstream pharmacological function. Evidence provenance is included because a mechanism demonstrated in a cellular or hepatic system should not be promoted automatically to a human solid-tumour mechanism.

Table 2 extends this comparison by summarising the relevant denominator, common surrogate readout, and stronger mechanistic evidence for each major step in the delivery sequence.

## 4. Lipid Nanomedicines Follow Distinct Delivery Routes

Mechanistic claims should remain bound to formulation class. Poly(ethylene glycol) (PEG)-modified liposomes, ionisable LNPs, permanently cationic systems, solid lipid nanoparticles (SLNs), nanostructured lipid carriers (NLCs), and lipid–polymer hybrids differ in internal structure, charge state, cargo organisation, lipid exchange, and release kinetics. Spherical nucleic-acid/LNP architectures further illustrate that nucleic-acid organisation and lipid presentation can differ substantially from conventional encapsulated LNPs [31]. A mechanism established in one class should therefore not be transferred uncritically to another.

Conventional liposomes often derive efficacy from prolonged circulation, altered tumour exposure and controlled small-molecule release without requiring evidence of cytosolic translocation of the intact carrier; Doxil illustrates both the value of this pharmacokinetic strategy and the importance of drug availability [22]. Ionisable LNPs instead protect nucleic acids during circulation and become more cationic as endosomal pH falls, promoting interactions with anionic lipids and non-bilayer phases that can support cytosolic delivery [1,8,32].

Permanently cationic systems can produce strong binding and high in vitro uptake, but fixed positive charge also promotes serum interactions, inflammatory toxicity and functionally ineffective sequestration after systemic administration [33]. SLNs and NLCs use solid or partially disordered lipid matrices suited to hydrophobic cargos and sustained release [34]. Lipid–polymer hybrids add independent core, shell and cargo disassembly kinetics, making multistage release itself a potential bottleneck [35].

“Endosomal escape efficiency”, “protein corona”, the “PEG dilemma”, and even “uptake pathway” are platform-conditioned concepts rather than universal constants. Cross-platform comparisons are most informative when cargos, biological models, doses, and functional endpoints are matched, and when mechanistic claims are tied to the specific biological route being tested. The principal mechanistic differences among these lipid nanomedicine classes are summarised in Table 3.

Although the present review focuses on lipid nanomedicines, its central principle—that upstream particle uptake should not be equated with productive delivery—also applies conceptually to polymer-based nanocarriers. However, polymer composition, degradation, cargo binding, release kinetics, and associated toxicity can introduce different rate-limiting steps; therefore, the mechanistic framework may be transferable, but quantitative efficiencies and platform-specific mechanisms require direct validation in polymeric systems [19,33,36].

## 5. From Synthetic Identity to Dynamic Biological Identity

The particle leaving the formulation vial is not necessarily the biological entity encountered by a receptor in vivo. Size, morphology, PEG density, ionisable-lipid chemistry, apparent pKa and helper lipids set the starting conditions, whereas plasma exposure drives lipid exchange and adsorption of proteins, lipoproteins and other biomolecules [27,36,37,38,39,40,41]. Biological identity therefore evolves but remains constrained by synthetic identity.

Apolipoprotein E (ApoE)-mediated hepatocyte targeting is a canonical example of endogenous targeting, but it is not a universal explanation for LNP biodistribution. Selective organ targeting (SORT) studies showed that a fifth lipid component can alter apparent pKa, corona composition and organ-selective functional mRNA delivery [42,43], while later work demonstrated ApoE-independent routes and roles for other proteins [27,39,40]. These predominantly hepatic data illustrate how biological identity can redirect functional cell allocation, not tumour delivery itself.

The classical hard “protein corona” concept also requires refinement for soft lipid particles. Photoaffinity chemoproteomics showed that conventional isolation can bias the proteins assigned to liposomes [38], while recent work highlighted contamination or co-isolation with extracellular vesicles, lipoproteins, and protein aggregates during LNP corona analysis [41,44]. For LNPs, the biologically relevant interface may involve adsorption, lipid exchange, lipoprotein association, and particle restructuring rather than a static shell. This distinction matters when targeting mechanisms are inferred from purified-corona proteomics. Corona state can also alter immune signalling rather than targeting alone, as illustrated by studies linking LNP corona formation to changes in NLR family pyrin domain containing 3 (NLRP3) inflammasome activation [45].

PEG stabilises particles and limits nonspecific interactions, yet persistent shielding can delay cell binding and membrane interactions. PEG-lipid anchor length and desorption therefore influence both corona evolution and entry [46]. The useful question is whether PEG residence time matches the required sequence of circulation, target-cell interaction and intracellular processing.

### Active Targeted Delivery with Ligand-Functionalized Lipid Carriers

Active ligands operate within this evolving interface. Their major benefit may be to alter cell-type allocation or internalisation after tissue access rather than to increase total tumour mass accumulation. Corona masking, colloidal effects and immune recognition can erode this benefit, so synthetic and endogenous targeting should be treated as interacting layers.

For ligand-functionalized lipid carriers, the success of receptor-mediated targeting additionally depends on whether the ligand remains accessible in vivo and whether the relevant receptor is sufficiently expressed on the therapeutically important cell population. Even when ligand-mediated binding or internalisation is enhanced, productive delivery can still fail downstream through limited tissue penetration, endosomal sequestration, recycling, or delayed cargo release. Therefore, evidence of successful active targeting should extend beyond increased ligand-dependent uptake to demonstrate preferential delivery to the intended cell population together with increased cargo-specific molecular action at an acceptable systemic exposure [36,47].

## 6. The Tumour–Nanoparticle Interface: Access Is Route- and Model-Dependent

Nanoparticle entry into solid tumours is constrained by systemic clearance, vascular architecture, endothelial transport, blood flow, interstitial pressure and extracellular matrix. Enhanced permeability and retention (EPR) is biologically real but heterogeneous, while active transport and retention can coexist with paracellular leakage [47]. The useful question is therefore which vascular route contributes to functionally relevant tumour access for a defined formulation in a defined tumour context.

Sindhwani et al. used three-dimensional electron microscopy and related methods to argue that most nanoparticle entry events in their tumour models were transcellular rather than through visible endothelial gaps [2]. The frequently cited value of up to ~97% is model- and material-specific. Subsequent studies identified specialised tumour endothelial populations [12], demonstrated albumin-nanoparticle transcytosis by intravital microscopy [14], and reported enhanced nanomedicine interactions with primary human breast-cancer-associated endothelial cells [13]. Together, these data establish endothelial biology as a design variable without defining a universal transcellular fraction [47].

The optimal particle for a leaky vessel is not necessarily optimal for receptor-mediated transcytosis. Size, shape, surface chemistry, protein association and dose can alter vascular contact and transport; saturation of hepatic clearance at high particle numbers can also increase tumour delivery [48]. Studies of transcytosis-oriented micelles and actively transported liposomes further show that transport mechanisms can alter extravasation, infiltration and tumour accumulation in formulation-specific ways [49,50]. Tumour access is therefore an emergent whole-organism property, not a fixed material constant.

After extravasation, matrix density, interstitial pressure and binding-site barriers can restrict penetration, while endothelial cells, macrophages and fibroblasts compete for material. Thus, whole-tumour delivery can increase without improving access to viable malignant cells. Tumour access is therefore meaningful only when a sufficient fraction of the administered material reaches the therapeutically relevant cell population. Figure 1 summarises this progression from systemic exposure and vascular crossing to tumour-interstitial access and allocation among competing tumour-associated cell populations.

## 7. Cellular Recognition and Internalisation: Entry Is Not Efficacy

At the target cell, membrane binding reflects the particle’s evolved biological identity together with the cell’s receptor landscape, glycocalyx, membrane composition, and endocytic state. Clathrin-dependent uptake, caveolar pathways, macropinocytosis, and phagocytosis can coexist and shift with cell phenotype and formulation [5,6,7]; a nanomedicine therefore rarely has one immutable uptake pathway.

Mechanistic assignment is weakened when it relies primarily on nonspecific pharmacological inhibitors. Chlorpromazine, dynasore, amiloride derivatives, and related agents can perturb trafficking, cytoskeletal dynamics, energetics, and compensatory uptake. A reduction in fluorescence can generate a pathway hypothesis, but stronger inference requires a more specific perturbation, orthogonal localization, and a functional delivery readout.

Faster entry is not necessarily better entry because endocytic routes differ in maturation, recycling and opportunities for membrane disruption. Rapid internalisation can funnel particles toward lysosomes, whereas slower routes may prolong residence in escape-permissive compartments. The relevant outcome is therefore not internalisation alone, but whether cellular entry is followed by favourable intracellular routing and, when required, cytosolic escape of functional cargo. These competing intracellular fates are illustrated schematically in Figure 2.

This principle reframes active targeting: ligand addition may leave total tumour accumulation unchanged while shifting which cells internalise the particle. Targeting should therefore be judged by cell-resolved functional delivery and target engagement rather than whole-tumour fluorescence or in vitro uptake alone.

## 8. Intracellular Attrition: Recycling and Lysosomal Sequestration

Internalisation begins rather than completes the intracellular problem. Early endosomes sort cargo toward recycling, maturation, lysosomes or other organelles; a particle entering the wrong branch can be pharmacologically lost despite strong intracellular fluorescence.

Sahay et al. showed that NPC1-dependent recycling can substantially limit siRNA-LNP delivery [6]. The often-quoted ~70% exocytosis estimate is instructive but specific to the studied systems, not a universal fraction. The broader lesson is that recycling can substantially offset gains achieved at the uptake step.

Lysosomal sequestration is a second sink: acidification and hydrolases can degrade cargo, alter lipid organisation and trap complexes. For nucleic acids, cytosolic release generally precedes terminal lysosomal accumulation; live-cell imaging shows that timing and organelle state can matter as much as total residence [7].

An attrition map is therefore more informative than a static colocalisation image. Quantifying the fractions that recycle, persist, degrade, escape, and remain competent for molecular action over time provides a route-specific estimate of intracellular loss and reveals whether a chemistry change improves favourable intracellular trafficking or simply feeds the same non-productive sink.

## 9. Endosomal Escape: Rare, Transient, and Easy to Mismeasure

Endosomal escape is a major bottleneck for many nucleic-acid LNPs [1]. Ionisable lipids become protonated during endosomal acidification and can promote non-bilayer structures, curvature and transient membrane defects. Escape is unlikely to be a single catastrophic event; depending on chemistry and cargo, it may involve transient pores, lipid mixing or localised membrane destabilisation [1,7,8].

Gilleron et al. estimated ~1–2% cytosolic escape of internalised siRNA in their experimental LNP system [5]. A 2026 in vivo Lysosomal Barcoding/LysoTag study estimated that ~8% of the assayed BiP-20 LNP material reached the cytosol within 30 min in liver [28]. These values should not be compared as universal efficiencies because formulation, organ, dose, denominator, time, and assay differ. Independent work further showed that LNP topology can regulate escape and cytoplasmic RNA delivery [8].

Measurement is itself a bottleneck. Galectin-8/9 recruitment reports endosomal membrane damage but does not by itself establish intact cargo translocation [7,9], while small or transient membrane defects may evade the same signature. Split-complementation, chloroalkane penetration assay (CAPA)-type methods, direct cytosolic tracking, and organelle isolation provide complementary views [10,17,18,28]. Escape efficiencies should therefore be interpreted as assay-defined, system-specific estimates rather than intrinsic material constants.

The relevant design variable is successful cytosolic escape: cytosolic entry of pharmacologically competent cargo without intolerable membrane injury or inflammatory sensing. Fusion-peptide LNPs illustrate ongoing attempts to improve this transition [20], while proposed post-escape aggregation and dissolution mechanisms identify additional potential sinks [51]. The objective is not maximal membrane disruption, but the highest safe downstream molecular action.

## 10. Cargo Release, Bioconversion, and Molecular Action Are Cargo-Specific Bottlenecks

Cytosolic arrival is not a universal endpoint. siRNA must become accessible to RISC, mRNA must remain intact and translatable, genome-editing systems may require coordinated delivery and nuclear access, and liposomal small molecules can depend mainly on extracellular or intracellular drug release rather than cytosolic translocation of intact carrier.

Carrier stability therefore has an optimum. Premature disassembly exposes cargo to degradation or off-target pharmacology, whereas excessive stability can retain cargo after cytosolic entry. Post-escape aggregation or delayed disassembly may add another sink [51], which is why release, activation where relevant and molecular action should remain explicit rather than being assumed consequences of escape.

Cargo-specific function separates presence from pharmacology: tissue mRNA abundance is not equivalent to protein expression in the intended cell, and biopsy siRNA is not equivalent to sequence-specific target cleavage. Functional barcoding likewise shows that particles with similar organ-level biodistribution can produce very different cell-level expression and biological outcomes [26,27].

Mechanistic endpoints should be cargo-specific: RISC loading or target cleavage for siRNA, cell-resolved protein translation for mRNA, verified on-target editing for genome-editing systems, and target-specific pharmacodynamics for small molecules [21,29,30].

## 11. What Constitutes Strong Mechanistic Evidence of Functional Delivery?

A mechanistic claim about functional delivery should address the following four related questions: where are the carrier and cargo; what biological or physicochemical process is required; does the cargo or cargo-derived active species execute its intended molecular action; and does the proposed mechanism persist at an appropriate level of biological complexity? Many studies resolve only localization, leaving a gap between observation, causality, function, and translational context.

Localization should distinguish surface association, internalisation, organelle residence, cytosolic access, and cargo release using orthogonal methods where possible. Causal inference is strengthened by specific perturbation, rescue, or an independent formulation-level test rather than by nonspecific uptake inhibitors alone. Functional evidence should be cargo-specific—for example, RISC loading or target cleavage for siRNA, cell-resolved protein translation for mRNA, verified on-target editing for genome-editing systems, or target-specific pharmacodynamics for small molecules. Finally, the proposed mechanism should be tested across increasing biological complexity, including human-relevant systems and in vivo cell attribution when feasible.

These forms of evidence are complementary rather than rank-ordered. Genetic perturbation is powerful when tractable, whereas redundant networks and physicochemical mechanisms may require rescue, orthogonal perturbation, or formulation-level controls. Human pharmacodynamics strengthens translational confidence but does not identify an intracellular route; rigorous cellular causality does not establish human exposure or therapeutic index. Claim language should therefore remain proportional to what the experiments actually demonstrate. Representative assays, their principal strengths and limitations, and useful complementary measurements are summarised in Table 4.

## 12. Model Dependence Is Mechanistic, Not a Nuisance Variable

A pathway measured in static two-dimensional (2D) culture is conditioned by that model. Sedimentation, serum composition, receptor abundance, cell cycle, absence of flow, immune competitors and extracellular matrix all alter nanoparticle–cell interactions, so the same formulation can behave differently across cell lines, primary cells, organoids and in vivo tissues [27,52].

Serum preparation illustrates this sensitivity: protein state can alter ApoE-mediated uptake of D-Lin-MC3-DMA (MC3)-containing LNPs [53], while PEG-lipid anchor chemistry and serum alter tracking and transfection kinetics [46]. Mechanistic studies should therefore report contextual conditions that can reshape the apparent pathway.

Spheroids and patient-derived organoids add architecture, gradients and multicellular organisation but lack full systemic pharmacokinetics and immune clearance. In vivo models add those constraints while introducing species-specific coronas, endothelium and immunity. Human endothelial studies [13] and clinical pharmacodynamics [21,54] are therefore valuable constraints on preclinical mechanisms.

The appropriate response is a staged mechanistic sequence: reductionist systems for causal dissection, three-dimensional (3D) systems for architecture and cellular competition, in vivo models for whole-organism constraints, and human data whenever feasible for translational validation. In vitro testing can establish whether a formulation improves a defined mechanistic step, but it cannot by itself determine the overall efficiency of a systemically administered drug-delivery system; such a conclusion requires validation in higher-complexity models and, where whole-organism delivery performance is claimed, in vivo evidence. Concordance across these levels is stronger evidence than performance in any one model alone.

## 13. Design Implication: Optimise the Bottleneck, Not the Brightest Signal

Nanomedicine design is a multi-objective optimisation problem: long circulation can conflict with cell interaction, strong membrane binding with colloidal stability, aggressive escape with tolerability, and stable complexation with timely release. These trade-offs should be tuned around the dominant bottleneck in a defined therapeutic context.

A practical optimisation sequence is to estimate where material is lost, identify the biological step with the greatest feasible safety-adjusted leverage, modify chemistry while measuring both the intended and downstream effects, and confirm the gain in a higher-complexity model. The numerically smallest fraction is not automatically the best target because achievable fold-change, coupling between processes, dose dependence, and toxicity determine whether a change improves the therapeutic outcome. This differs from ranking reporter intensity first and assigning mechanism retrospectively.

Chemical structure can alter several terms simultaneously. Ionisable lipid tails, head groups and linkers affect morphology, pKa, protein association, membrane interaction and biodegradation [8,32,55]; helper lipids and cholesterol analogues alter structure and cell performance [52]; PEG anchors tune stability and shedding [46]; and SORT lipids alter organ targeting [42,43]. Such pleiotropy limits simple structure-activity extrapolation.

High-throughput in vivo barcoding and functional mRNA screens shift selection toward downstream functional endpoints [26], while chemistry-guided studies increasingly tune delivery beyond the liver [56]. These approaches should still distinguish tissue abundance from cell-specific expression and be followed by causal deconvolution. Data-driven or machine-learning approaches may eventually capture nonlinear interactions among formulation variables, but only if trained on mechanistically resolved endpoints rather than reporter intensity alone.

## 14. Translational Case Studies: Delivery Mechanisms and the Therapeutic Index

Clinical experience is the strongest stress test of whether a mechanistic delivery narrative captures the therapeutic index. The examples below are illustrative rather than exhaustive; they were selected because they highlight different biological bottlenecks and different strengths or gaps in the available evidence. Table 5 therefore reports evidence provenance, mechanistic interpretation, and unresolved questions rather than assigning a numerical score.

ALN-VSP. In a first-in-human LNP trial, siRNA was detected in tumour biopsies and RNAi-specific VEGF cleavage was demonstrated in liver-containing biopsy samples, with pharmacodynamic and antitumour activity in some patients [21]. The strongest 5′-RACE signals arose mainly from normal liver, however. ALN-VSP therefore provides rare human evidence of cargo-specific RNAi action but does not establish tumour-cell target engagement or the intracellular route; the evidence for molecular action and human translation is stronger than the evidence for tumour-cell attribution or compartment-resolved trafficking.

MRX34. The liposomal miR-34a mimic showed biological activity in advanced solid tumours, but development stopped after severe immune-mediated toxicities, including four deaths in expansion cohorts [54]. The case shows that intracellular delivery and pharmacological activity cannot rescue an unacceptable therapeutic index: cargo biology, immune sensing, dose, exposure and formulation toxicity remain parallel constraints.

Doxil/Caelyx. PEGylated liposomal doxorubicin illustrates a different value proposition: altered pharmacokinetics and toxicity with therapeutic activity not dependent on demonstrating cytosolic escape of the intact carrier. Tumour accumulation and drug availability remain separable [22], and randomised comparisons often show clearer tolerability advantages than efficacy gains [23]. For small-molecule liposomes, clinical performance may therefore be governed primarily by appropriately timed active-drug exposure.

Nanoliposomal irinotecan (nal-IRI/Onivyde). Irinotecan efficacy depends on formation and persistence of SN-38; preclinical work showed that tumour deposition and intratumour prodrug conversion are critical determinants of SN-38 exposure and response [24]. NAPOLI-1 demonstrated a survival benefit with nal-IRI plus fluorouracil/folinic acid [25]. The relevant sequence is therefore tumour deposition, local bioconversion and sustained active-drug exposure rather than intact liposome-to-cytosol delivery.

Together, these cases argue against a single success metric for nanomedicine. Human translation requires alignment of organ tropism, cell allocation, cargo-specific requirements, exposure–response, and safety. The clinically useful question is which biological processes convert the administered formulation into the relevant active therapeutic species, in which cell or compartment, at what dose, and at what systemic cost.

## 15. Discussion: Major Controversies Reframed as Falsifiable Questions

EPR versus transcytosis. The relevant question is not which mechanism “wins” globally, but what fraction of a defined formulation crosses a defined tumour vasculature by each route at a clinically relevant exposure, and whether that route contributes to the intended cell population. Sindhwani et al. shifted attention toward transcellular transport [2], while other transport-oriented studies reinforce the importance of endothelial biology [12,13,14,49,50]. Model- and material-specific quantification—and ultimately human vessel-resolved measurements—remain necessary.

Is there an optimal uptake pathway? Probably not universally. The falsifiable formulation-specific question is whether perturbing pathway X changes cytosolic cargo and target engagement after controlling for total uptake. This directly separates entry from functionally useful intracellular routing.

Does active targeting improve delivery? Whole-tumour accumulation and target-cell delivery are distinct endpoints. A ligand may be valuable without increasing percentage injected dose per gram (%ID/g) if it redirects accessed dose toward the intended cell and increases target engagement without accelerating clearance. Conversely, excessive avidity can impair tissue penetration. The appropriate endpoint is therefore cell-resolved pharmacological output rather than bulk tumour signal.

Is endosomal escape always dominant? It is a major limitation for many nucleic-acid LNPs, but the dominant bottleneck can shift to extrahepatic access, cell allocation, cargo release, translation, or immune toxicity. As individual barriers are improved, another step may become limiting; this argues against assuming one universally rate-limiting process.

Are escape efficiencies comparable across studies? Not without harmonised assays, reference formulations, matched cargo and dose, and an orthogonal functional endpoint. Galectin recruitment, split-complementation, CAPA-type assays, and lysosomal barcoding interrogate different events and time windows; the ~1–2% and ~8% values are system-specific calibrations rather than competing universal truths [5,9,10,17,28].

What is a protein corona in an LNP? Soft lipid particles can exchange lipids, associate or fuse with lipoproteins and co-isolate with extracellular vesicles, complicating classical corona proteomics [39,41]. The falsifiable goal is to identify which molecular associations are physically present on the circulating particle and causally required for cell-specific functional delivery. Quantitative delivery values should be interpreted according to the biological transition and denominator being measured. For example, a widely cited cross-platform analysis found that a median of approximately 0.7% of the administered nanoparticle dose reached solid tumours [4]. This tumour-level estimate does not indicate the fraction reaching the intended tumour cell or intracellular target. Downstream measurements of recycling, cytosolic escape, or vascular transport therefore represent distinct efficiencies and should not be interpreted as interchangeable measures of overall delivery. Selected quantitative estimates discussed throughout this review, together with the experimental contexts that limit their generalisation, are summarised in Table 6.

## 16. Practical Reporting Considerations for Mechanistic Delivery Studies

The mechanistic themes summarised above support several practical reporting considerations. These are intended to improve interpretability and claim calibration and are not intended as a consensus reporting standard:

1. Define the material in the relevant biological state. Report composition, size distribution, morphology, encapsulation, apparent pKa where relevant, stability, PEG-lipid behaviour, and characterisation in biologically relevant fluids rather than buffer alone.
2. Separate exposure, association, and internalisation. Use extracellular quenching, acid washing, or equivalent controls and quantify cell-type allocation under conditions that reflect relevant flow, serum, and cellular competition.
3. Resolve trafficking and attrition dynamically. Measure recycling, lysosomal accumulation, and temporal organelle transitions rather than relying on a single static colocalisation endpoint.
4. Quantify cytosolic escape and cargo release when these steps are biologically required. Pair membrane-damage reporters with a direct cytosolic-delivery assay and, where possible, independently track carrier and cargo to demonstrate dissociation.
5. Establish causality and cargo-specific molecular action. Perturb the proposed pathway with the most specific feasible method, perform rescue or orthogonal confirmation where appropriate, and show that the perturbation changes a cargo-specific pharmacological endpoint. For prodrugs or bioconversion-dependent systems, quantify the relevant active species and its target engagement.
6. Test the mechanism at increasing biological complexity. Progress from reductionist cells to 3D or human-relevant models, in vivo cell-resolved pharmacology, and ultimately translational pharmacodynamics when feasible.

A study does not need to address every consideration to be informative, but the language of its mechanistic claim should be proportional to the evidence generated. A formulation that increases total cellular fluorescence, for example, can reasonably be described as increasing association or exposure; it should not be described as improving functional delivery unless downstream compartmental and pharmacological evidence supports that inference.

## 17. Limitations of the Narrative Review and Mechanistic Interpretation

This narrative synthesis is intended to organise mechanistic evidence rather than to provide a predictive pharmacokinetic model or a systematic meta-analysis. Delivery efficiencies vary with dose, time, formulation, cargo, assay, cell state, tumour type, and biological context, and the dominant bottleneck cannot be identified without direct measurement. Relevant biological routes can branch, recycle, and converge, so mechanistic conclusions should remain specific to the formulation and experimental system in which they were demonstrated.

Measurement remains the principal practical limitation. Absolute cytosolic cargo concentrations, cell-specific denominators, and time-resolved carrier–cargo dissociation are unavailable for many systems, and labels can report carrier persistence after cargo release or degradation. Quantitative efficiencies from unrelated formulations, organs, doses, or assays should therefore not be combined as though they describe a single biological route. The immediate value of mechanistic analysis is to expose missing measurements, compartmental ambiguities, and mismatches between upstream surrogate signals and downstream function.

The review is deliberately scoped to systemically administered lipid nanomedicines in solid tumours, with hepatic and non-oncology LNP studies used selectively when they establish general principles of biological identity, intracellular routing, endosomal escape, or functional delivery. Those mechanisms may not persist under tumour-specific vascular, stromal, immune, or cellular constraints. Because this is a narrative review, study selection is purposive and mechanism-centred rather than exhaustive; conclusions should therefore be read as an interpretive synthesis of the cited evidence rather than as pooled estimates of effect.

## 18. Future Directions and Research Priorities

First, the field needs more absolute or near-absolute measurements of cytosolic cargo in vivo. Advances in split-complementation, live-cell reporters, single-molecule imaging, CAPA-type assays, and Lysosomal Barcoding now make it possible to move beyond qualitative puncta [9,10,17,18,28]. Standard reference formulations, matched cargos, and time-resolved sampling could enable cross-laboratory benchmarking of escape estimates and measurement bias.

Second, tumour delivery should be resolved as a cell-allocation problem. Intravital microscopy, spatial transcriptomics, imaging mass cytometry and single-cell sequencing can connect particle location with cell state, allowing investigators to determine whether hypoxic cancer cells, inflammatory macrophages, fibroblasts or endothelial cells receive functionally active cargo. The important metric is not only how much material reaches the tumour, but which cellular subpopulation converts it into pharmacology.

Third, the biological identity of LNPs should be measured dynamically and with methods tailored to soft nanoparticles. Proteomics should distinguish genuine particle-associated proteins from lipoproteins, extracellular vesicles and purification artefacts [38,39,41]. Causal depletion/add-back experiments are needed to show that candidate corona components actually control functional delivery.

Fourth, formulation screening should use functional barcodes and causal follow-up. High-throughput in vivo screens can identify rare particles with extrahepatic tropism [26], but mechanistic interpretation should then identify the biological step responsible for the gain. This combination—functional discovery followed by causal deconvolution—is more likely to generate transferable design rules than reporter-only screening.

Fifth, endosomal escape should be optimised together with post-escape release and tolerability. Stronger membrane disruption is not automatically better if it produces organelle stress, inflammatory signalling or tissue injury, and efficient membrane crossing can still fail if cargo remains aggregated or carrier-bound [51]. The relevant objective function is cargo-specific molecular action per tolerated dose. New lipids, fusion peptides and degradable chemistries should therefore be benchmarked by cytosolic delivery, cargo release, cargo-specific function and safety [20,28].

Finally, human translation should enter mechanism studies earlier. Primary human endothelial cells, patient-derived organoids, ex vivo tumour slices and opportunistic clinical biopsies can reveal species-specific assumptions before large efficacy trials. In oncology, where the target tissue is heterogeneous and extrahepatic, early human-relevant testing may be essential to break the recurrent cycle of compelling mouse biodistribution followed by weak human pharmacology.

## 19. Conclusions

The central lesson is that tumour accumulation, cell association, internalisation, endosomal membrane damage, cytosolic availability, cargo release, bioconversion where required, and molecular action are experimentally distinct states. Treating all of them as “delivery” obscures the attrition points that formulation design is intended to solve.

Across the evidence reviewed here, successful lipid nanomedicine delivery depends on the alignment of the following multiple established biological processes: tumour access, allocation to the therapeutically relevant cell population, intracellular routing, endosomal escape when required, cargo release or bioconversion, and cargo-specific molecular action. The importance of each process varies with platform and payload. Studies of siRNA-LNP escape and recycling, tumour vascular transport, clinical RNA delivery, liposomal doxorubicin, and nanoliposomal irinotecan all show that an upstream gain is meaningful only when it is preserved through the downstream steps required for pharmacology.

The resulting design rule is not to dismiss uptake, tumour accumulation, EPR, active endothelial transport, targeting ligands, or biomolecular coronas, but to place each observation in the correct causal position and ask whether an apparent gain survives downstream, remains cell-specific, and is achieved at acceptable systemic cost. For mechanistic studies, the preferred endpoint is evidence-proportionate, cell-resolved molecular action; for translation, it is safe pharmacological output at a clinically relevant exposure.

## Figures and Tables

**Figure 1:**
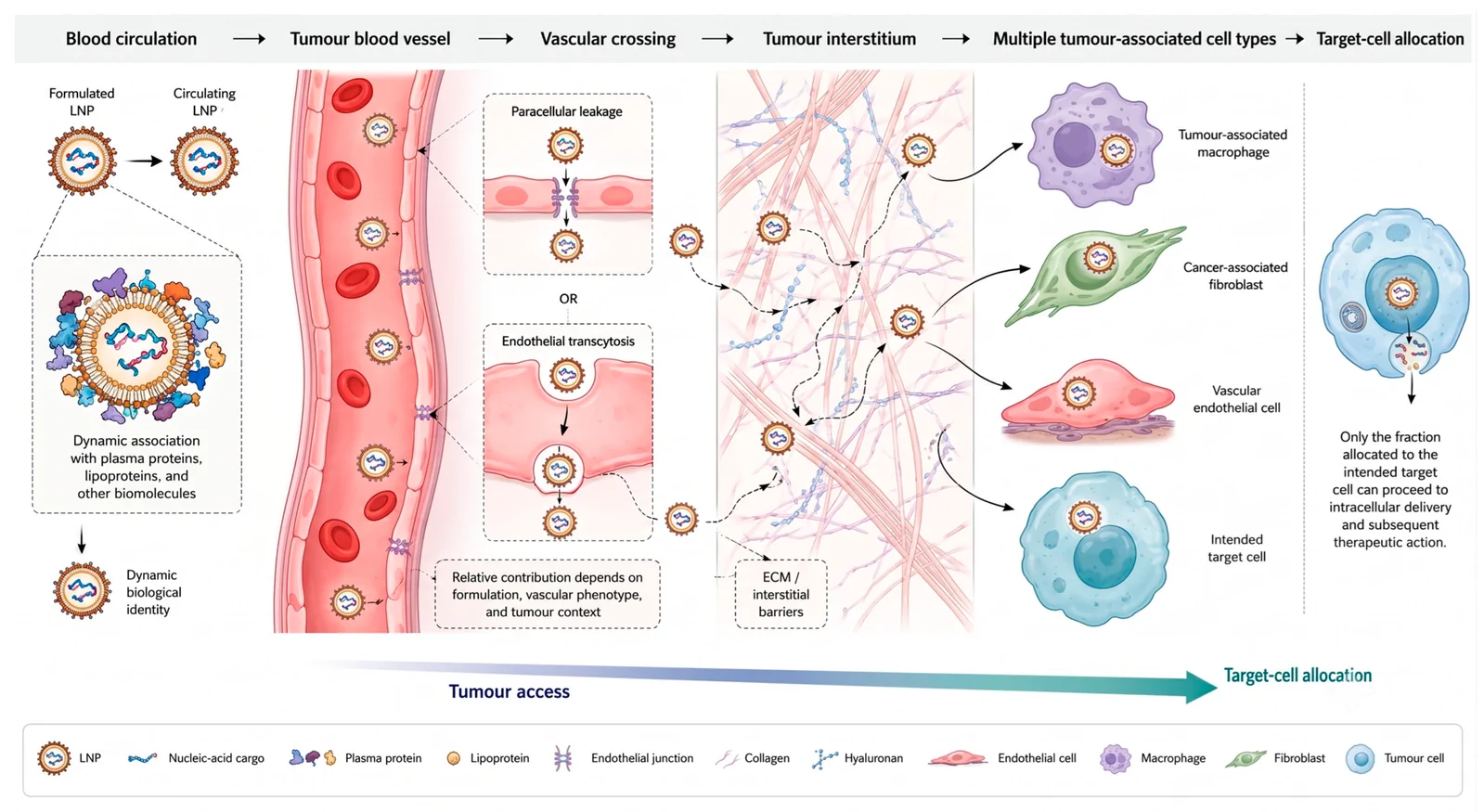
Tumour–nanoparticle interface and target-cell allocation. Following systemic administration, the synthetic identity of a lipid nanoparticle evolves through dynamic interactions with plasma proteins, lipoproteins, and other circulating biomolecules, generating a context-dependent biological identity. Tumour access may occur through formulation- and model-dependent vascular routes, including paracellular leakage and endothelial transcytosis, whose relative contributions can vary with vascular phenotype and tumour context. After extravasation, extracellular-matrix and interstitial barriers can restrict penetration, while endothelial cells, tumour-associated macrophages, fibroblasts, and other tumour-associated cell populations compete for accessible nanoparticle material. Consequently, whole-tumour accumulation does not establish delivery to the intended target-cell population. Only the fraction allocated to the intended target cell can proceed to subsequent intracellular delivery steps. The schematic is conceptual; the relative contributions of vascular transport routes, stromal barriers, and competing cell populations are formulation-, tumour-, dose-, and model-dependent.

**Figure 2:**
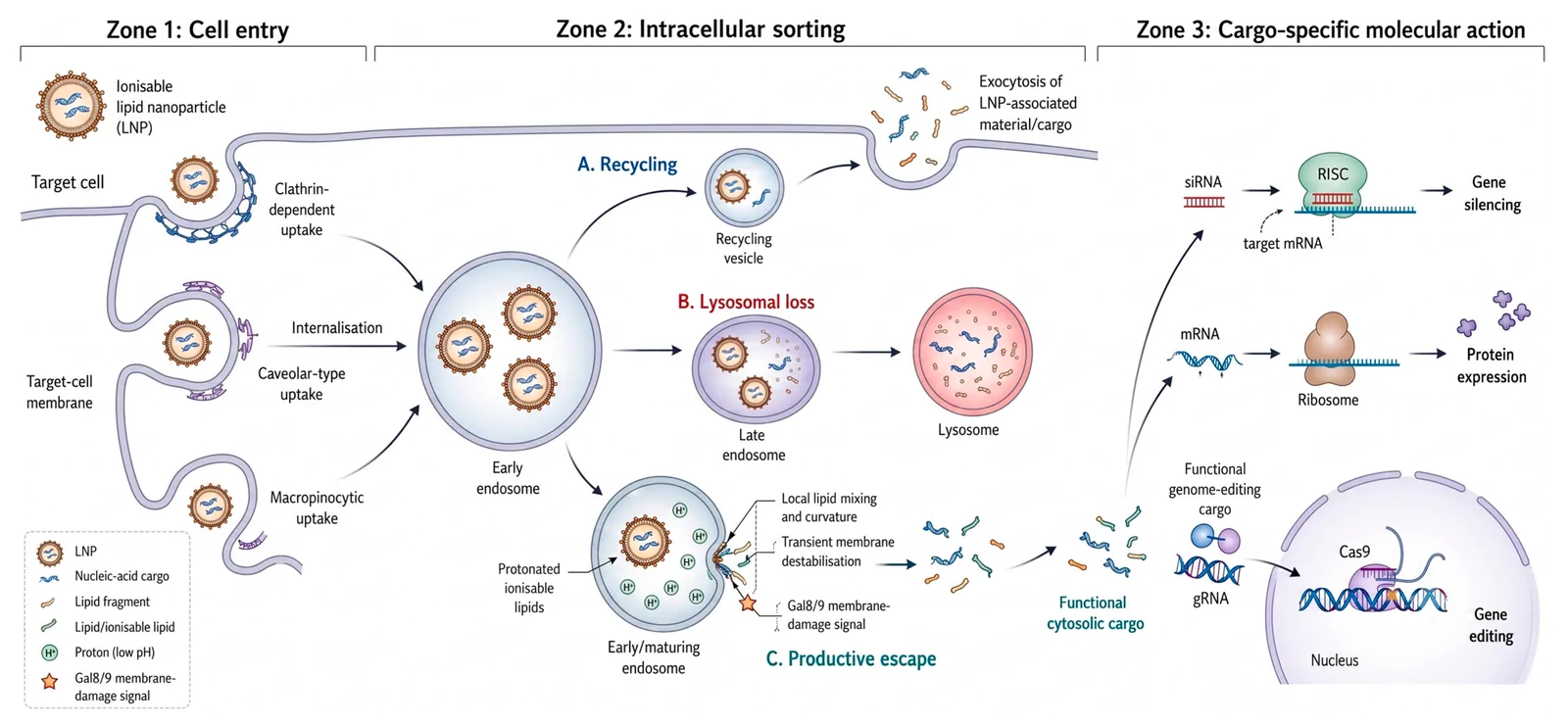
Intracellular fate of lipid nanomedicines. Following cellular internalisation, lipid nanoparticle cargo can undergo recycling/exocytosis, endosomal maturation and lysosomal sequestration, or productive endosomal escape. Escaped nucleic-acid cargo must subsequently remain pharmacologically competent to generate cargo-specific molecular action, including siRNA-mediated gene silencing, mRNA translation, or genome editing. The illustrated escape mechanism is conceptual and may involve formulation-dependent protonation of ionisable lipids, local lipid mixing, membrane curvature, and transient membrane destabilisation. Galectin-8/9 is shown as a membrane-damage signal and does not by itself establish cytosolic translocation of intact cargo. The pathways are schematic and formulation-, cargo-, cell-, and context-dependent.

**Table 1:** Published examples illustrating divergence between upstream delivery readouts and downstream mechanism or pharmacological function.

Published Example	Upstream Observation	Downstream Mechanistic or Pharmacological Evidence	How the Interpretation Changes	Evidence Provenance
Gilleron et al. [5]	siRNA-LNP internalisation	Only ~1–2% of internalised siRNA reached the cytosol.	Uptake does not indicate cytosolic delivery.	Cellular and in vivo hepatic mechanistic evidence
Sahay et al. [6]	High siRNA-LNP internalisation	~70% of internalised siRNA underwent recycling/exocytosis; NPC1 regulated this process.	Recycling can substantially reduce productive delivery after uptake.	Cellular mechanistic evidence
Sindhwani et al. [2] and related transport studies [12,14]	Tumour accumulation and vascular association	Endothelial transcellular transport contributed to nanoparticle entry in selected tumour models.	Tumour accumulation does not establish the vascular entry route.	Preclinical solid-tumour evidence; model dependent
ALN-VSP [21]	siRNA detected in human biopsies	Vascular endothelial growth factor (VEGF) messenger RNA (mRNA) cleavage was demonstrated, strongest in liver-containing biopsies; pharmacodynamic signals of target downregulation were also observed.	Tissue detection does not establish tumour-cell-specific delivery.	Human phase I evidence
Doxil/Caelyx [22,23]	Prolonged circulation and altered drug exposure	Released doxorubicin becomes available to tumour cells; clinical evidence shows altered pharmacokinetics (PK) and improved tolerability.	For this platform, active-drug exposure is more informative than intact-carrier cytosolic entry.	Clinical pharmacological and therapeutic evidence
Nanoliposomal irinotecan [24,25]	Prolonged tumour exposure	Tumour deposition and local conversion to SN-38 prolong active exposure; phase III survival benefit was shown for nanoliposomal irinotecan (nal-IRI) + 5-fluorouracil/leucovorin (5-FU/LV).	Active-metabolite exposure can be the relevant downstream endpoint.	Preclinical mechanistic plus phase III evidence

**Table 2:** Mechanistic readouts, relevant denominators, common surrogates, and stronger evidence.

Mechanistic Step	Relevant Denominator and Measured Transition	Common Surrogate	Why the Surrogate Can fail	Stronger Mechanistic Evidence
Tumour access	Administered cargo (absolute access) or cargo presented to tumour vasculature (vessel-crossing analysis) → spatially resolved extravascular tumour cargo.	Whole-tumour fluorescence; percentage injected dose per gram (%ID/g)	Includes intravascular, perivascular and stromal sequestration [2,12,13,14].	Spatially resolved extravascular quantification with explicit vessel exclusion; pair with cell-type allocation [2,12,13,14].
Target-cell allocation	Extravascular tumour-accessed cargo → allocated to the defined therapeutically relevant cell population.	Tumour accumulation	Does not identify malignant versus endothelial, myeloid or fibroblast uptake [12,26].	Cell-resolved in vivo biodistribution, ideally paired with a cargo-specific functional readout [12,26,27].
Internalisation	Cargo associated with the defined target-cell population → intracellular cargo after exclusion of extracellular or surface-bound material.	Total cell fluorescence	Cannot exclude surface-bound material [5,6,7].	Extracellular quenching/acid wash plus 3D imaging or ultrastructural confirmation [5,6,7].
Intracellular routing	Internalised cargo in the defined target cell → cargo entering the specified intracellular trafficking branch.	Colocalisation with a single endosomal marker	Static colocalisation cannot resolve dynamic sorting or recycling [5,6,7].	Live-cell tracking with endosomal, recycling and lysosomal markers plus causal perturbation when feasible [5,6,7].
Endosomal escape	Cargo in the specified pre-escape endosomal pool → intact or pharmacologically competent cargo detected in the cytosol; denominator and time window should be stated.	Galectin puncta or membrane damage	Damage does not prove intact cargo entered the cytosol [9,10]	Split-complementation, CAPA-type assays, direct cytosolic tracking, or organelle-based quantification [10,17,18,28].
Cargo release/bioconversion	Cargo reaching the compartment in which release or activation can occur → released cargo or the relevant activated/bioconverted therapeutic species.	Carrier signal or total cargo abundance	Carrier signal may not report released cargo; total cargo may be degraded, inactive, or unconverted [22,24].	Independent carrier/cargo tracking plus intact-cargo, released-cargo, or active-species measurement [22,24].
Cargo-specific molecular action	Pharmacologically competent cargo or cargo-derived active species in the relevant cell/compartment → defined cargo-specific molecular action or target engagement.	Cytosolic cargo or tissue cargo abundance	Presence does not establish cargo-specific function in the intended cell [21,26,29,30]	RISC loading/target cleavage, cell-resolved protein expression, verified on-target editing, or target-specific pharmacodynamics [21,26,29,30].

**Table 3:** Selected lipid nanomedicine classes and mechanistic consequences.

Platform	Representative Architecture	Typical Cargo	Dominant Design Advantage	Principal Mechanistic Limitation	Interpretive Caution
PEGylated liposome	Stable phospholipid/cholesterol bilayer; aqueous core	Small molecules	Long circulation and altered tissue PK [22]	Slow or spatially mismatched drug release [22]	Tumour accumulation need not predict free-drug exposure [22]
Ionisable LNP	Dynamic lipid–nucleic-acid assembly; near-neutral at physiological pH	siRNA, mRNA, gene-editing RNA	Protection plus pH-responsive membrane interaction [1,8,32]	Recycling, lysosomal loss and inefficient cytosolic release [5,6,7,8]	Escape values are cargo/formulation/model dependent [5,8,28]
Permanently cationic lipid system	Fixed positive surface charge	DNA/RNA	Strong binding and cellular association	Opsonisation, toxicity, lysosomal trapping [16,33]	In vitro uptake can overpredict in vivo performance [16,33]
SLN/NLC	Solid or partially disordered lipid matrix	Hydrophobic small molecules	Sustained release and physical stability [34]	Release kinetics and matrix design can constrain active-drug availability [34]	Evidence is strongest for hydrophobic small-molecule delivery; release is formulation-specific [34]
Lipid–polymer hybrid	Polymeric core with lipid shell	Small molecules, RNA, combinations	Independent tuning of stability and surface biology [35]	Multistage disassembly may become rate-limiting [35]	Mechanism depends on which component controls release [35]

**Table 4:** Assays for uptake, trafficking, cytosolic access, and cargo-specific function.

Method	Primary Information	Major Strength	What It Does Not Prove	Recommended Complement
Flow cytometry of labelled carrier/cargo	Cell-associated signal [7]	High throughput [7]	Does not by itself establish internalisation or cytosolic delivery [7]	Surface-stripping or quenching control + functional readout [7]
Confocal z-stacks/live-cell imaging	Spatial localization; temporal trafficking when acquired live [5,7]	3D compartmental context; dynamic trafficking with live-cell acquisition [5,7]	Absolute cytosolic concentration or cargo-specific function [5,7]	Direct cytosolic-access assay + cargo-specific functional endpoint [5,7,10]
Electron microscopy	Ultrastructural localization [5]	Direct organelle context [5]	Dynamic kinetics or cargo function [5]	Live-cell imaging + pharmacology [5,6]
Galectin-8/9 (Gal8/Gal9) recruitment	Endosomal membrane damage [7,9]	Sensitive spatiotemporal reporter [7,9]	Intact cargo translocation [7,9,10]	Split-complementation or direct cytosolic assay [10]
Split luciferase/split protein	Cytosolic access of tagged cargo/reporters [10]	Direct cytosolic-access readout [10]	Cargo-specific target engagement [10]	Cargo-specific functional endpoint [10,21,29,30]
CAPA-like cytosolic-access assay	Cytosolic access of chloroalkane-tagged cargo [17]	Quantitative cell-based readout of cytosolic penetration [17]	Native untagged-cargo behaviour, organellar history, or target engagement [17]	Orthogonal trafficking/localization + cargo-specific functional endpoint [17]
Gene-editing/siRNA perturbation of trafficking genes	Causal biological requirement [6]	Mechanistic specificity [6]	Whether the effect is direct without orthogonal localization [6]	Rescue experiment + trafficking + functional endpoint [6]
Cargo-specific functional readout/pharmacodynamics	Target engagement/function [21,29,30]	Direct target-engagement evidence [21,29,30]	Exact route taken by carrier [21,29,30]	Pair with trafficking and causal perturbation [21,29,30]
Lysosomal barcoding/organelle immunoisolation [28]	In vivo endolysosomal retention and escape kinetics [28]	Directly links organelle-associated cargo to time-resolved in vivo escape estimates [28]	Universal escape efficiency or extrahepatic generalizability [28]	Cell-resolved pharmacology + orthogonal trafficking/escape assay [28]

**Table 5:** Translational examples: mechanistic bottlenecks, evidence strengths, and unresolved questions.

System	Mechanistic Pathway or Bottleneck	Evidence Provenance	Evidence Strengths and Unresolved Questions	Translational Lesson
ALN-VSP	Tumour/organ exposure → target-cell attribution unresolved → RNAi-specific molecular action [21]	Human first-in-human trial [21]	Strength: RNAi-specific molecular action and human context. Gap: tumour-cell attribution and intracellular route were not resolved [21].	Human molecular action is stronger evidence than bulk biopsy abundance, but it does not by itself prove functional delivery to tumour cells [21].
MRX34	Cellular delivery and miRNA activity constrained by immune toxicity [54]	Human phase I trial [54]	Strength: human biological activity and safety context. Gap: intracellular route and cell-specific target engagement were incompletely defined [54].	Evidence of pharmacological activity is insufficient if immune toxicity collapses the therapeutic index [54].
Doxil/Caelyx	Tumour exposure → drug release/availability → active-drug exposure → target engagement [22,23]	Clinical pharmacokinetic/therapeutic experience [22,23]	Strength: human PK, safety, and pharmacological outcomes. Interpretation: carrier-level cytosolic escape need not be the relevant success criterion for this platform [22,23].	For small-molecule liposomes, timed drug release and active-drug exposure can dominate the therapeutic pathway [22,23].
SORT LNPs	Biological identity → organ/cell allocation → functional expression [27,40,42,43]	Preclinical, predominantly hepatic evidence [27,40,42,43]	Strength: functional expression with mechanistic receptor/corona studies. Gap: direct solid-tumour generalizability [27,40,42,43].	Biodistribution and functional transfection are separable; endogenous targeting can be engineered, but provenance limits tumour claims [27,40,42,43].
Nanoliposomal irinotecan (nal-IRI/Onivyde)	Tumour deposition → local bioconversion to SN-38 → sustained active-drug exposure [24]	Preclinical solid-tumour evidence plus phase III clinical efficacy [24,25]	Strength: tumour deposition and SN-38 conversion evidence; human outcome. Gap: fine cell-level attribution remains less resolved [24,25].	Clinical benefit may depend on a cargo-derived active species and duration of active-drug exposure rather than carrier cytosolic entry [24,25].

**Table 6:** Reported quantitative estimates in lipid nanomedicine delivery and why they are context-dependent.

Reported Estimate	Biological Event Being Estimated	System, Assay and Time Context	Mechanistic Significance	Why It is Not a Universal Constant
~1–2% endosomal escape [5]	Fraction of internalised siRNA estimated to reach the cytosol [5]	In vitro cellular and mouse-liver siRNA-LNP systems; quantitative imaging and electron microscopy [5]	Illustrates intracellular escape as a severe attrition step [5]	Specific formulation, cargo, cell model, time window and analytical definition of escape [5]
~70% recycling/exocytosis [6]	Large fraction of internalised siRNA-LNP material returned to the extracellular space [6]	Studied siRNA-LNP/cell systems; NPC1-dependent trafficking and recycling measurements [6]	Demonstrates that high internalisation can be substantially offset by a downstream recycling sink [6]	Magnitude depends on formulation, cell state, dose and observation window [6]
Up to ~97% transcellular entry [2]	Fraction of observed nanoparticle entry events assigned to endothelial transcellular processes rather than visible gaps [2]	Selected nanoparticle/tumour models; three-dimensional electron microscopy and related analyses [2]	Shifted attention from an EPR-only model toward active endothelial transport [2]	Event-based estimate in specific materials and tumour models; not a universal fraction for human tumours [2]
~8% cytosolic material within 30 min [28]	Fraction of assayed BiP-20 LNP material estimated to reach the cytosol in the hepatic system [28]	Branched ionisable LNP in liver; in vivo Lysosomal Barcoding/LysoTag at an early time point [28]	Shows that escape can be measured dynamically in vivo and can differ from classic in vitro estimates [28]	Hepatic, formulation-, assay-, denominator-, and time-window-specific calibration [28]

## Data Availability

No new data were created or analysed in this study. Data sharing is not applicable to this article.
